# Manipulating the tumor immune microenvironment to improve cancer immunotherapy: IGF1R, a promising target

**DOI:** 10.3389/fimmu.2024.1356321

**Published:** 2024-02-14

**Authors:** Marsha Pellegrino, Valerio Secli, Silvia D’Amico, Lucia Lisa Petrilli, Matteo Caforio, Valentina Folgiero, Nicola Tumino, Paola Vacca, Maria Vinci, Doriana Fruci, Emmanuel de Billy

**Affiliations:** ^1^ Oncohematology and Pharmaceutical Factory Research Area, Pediatric Cancer Genetics and Epigenetics Unit, Bambino Gesù Children’s Hospital-IRCCS, Rome, Italy; ^2^ Immunology Research Area, Innate Lymphoid Cells Unit, Bambino Gesù Children’s Hospital-IRCCS, Rome, Italy

**Keywords:** immuno-oncotherapy, tumor microenvironment, IGF1R, cancer immunity, immunomodulation

## Abstract

Cancer immunotherapy has made impressive advances in improving the outcome of patients affected by malignant diseases. Nonetheless, some limitations still need to be tackled to more efficiently and safely treat patients, in particular for those affected by solid tumors. One of the limitations is related to the immunosuppressive tumor microenvironment (TME), which impairs anti-tumor immunity. Efforts to identify targets able to turn the TME into a milieu more auspicious to current immuno-oncotherapy is a real challenge due to the high redundancy of the mechanisms involved. However, the insulin-like growth factor 1 receptor (IGF1R), an attractive drug target for cancer therapy, is emerging as an important immunomodulator and regulator of key immune cell functions. Here, after briefly summarizing the IGF1R signaling pathway in cancer, we review its role in regulating immune cells function and activity, and discuss IGF1R as a promising target to improve anti-cancer immunotherapy.

## 1 Introduction

Cancer immunotherapy consists in stimulating or manipulating specialized cells of the immune system to boost their killing activity against malignant cells. Monoclonal antibodies to target tumor-associated antigens or immune checkpoint (IC) molecules, anti-cancer vaccines, small chemicals that boost the intrinsic activity of immune cells or the adoptive transfer of specialized and engineered immune cells, are different immunotherapeutic strategies in development for the treatment of cancer. While some of these approaches have been successful for hematological tumors, they have, so far, demonstrated only limited efficacy in patients affected by solid tumors (1).

One of the major factors limiting the efficacy of these immunotherapeutic approaches is related to the tumor microenvironment (TME) (2). The TME promotes tumor progression in part by maintaining an immunosuppressive state. The suppressive immune TME is the result of the continuous crosstalk between the tumor cells and the different cell subsets of the immune system. This complex and redundant crosstalk occurs mainly through direct cell-cell contacts as well as via the release of secreted factors, including metabolites, cytokines, chemokines, and growth factors, which stimulate the recruitment of immunosuppressive cells and impair the infiltration and activation of immune cells with anti-tumor activity (3). Therefore, the identification of targetable factors able to turn the immunosuppressive TME into a more favorable environment for anti-cancer immunity will certainly be beneficial for the improvement of cancer immunotherapy.

Among the growth factors, the insulin-like growth factor 1 (IGF1) is well known for its involvement in tumorigenesis, metastasis and drug resistance (4). The axis represented by IGF1 and its receptor, the insulin-like growth factor 1 receptor (IGF1R), is deregulated in many cancer cell types (5). In addition to its direct involvement in cancer cell survival and proliferation, the IGF1/IGF1R axis is also appearing as a key factor involved in the regulation of immunity and may be an important player in modulating the immune-compartment of the TME, and thus, the cancer-related immune response.

In this paper, after a brief description of the main signaling pathways downstream of IGF1R, we review the latest advances related to the role of the IGF1/IGF1R axis in immune cell regulation and discuss the potential of targeting IGF1R as a strategy to improve the efficacy of current cancer-immunotherapy approaches.

## 2 The IGF1R signaling pathway

**Figure 1:**
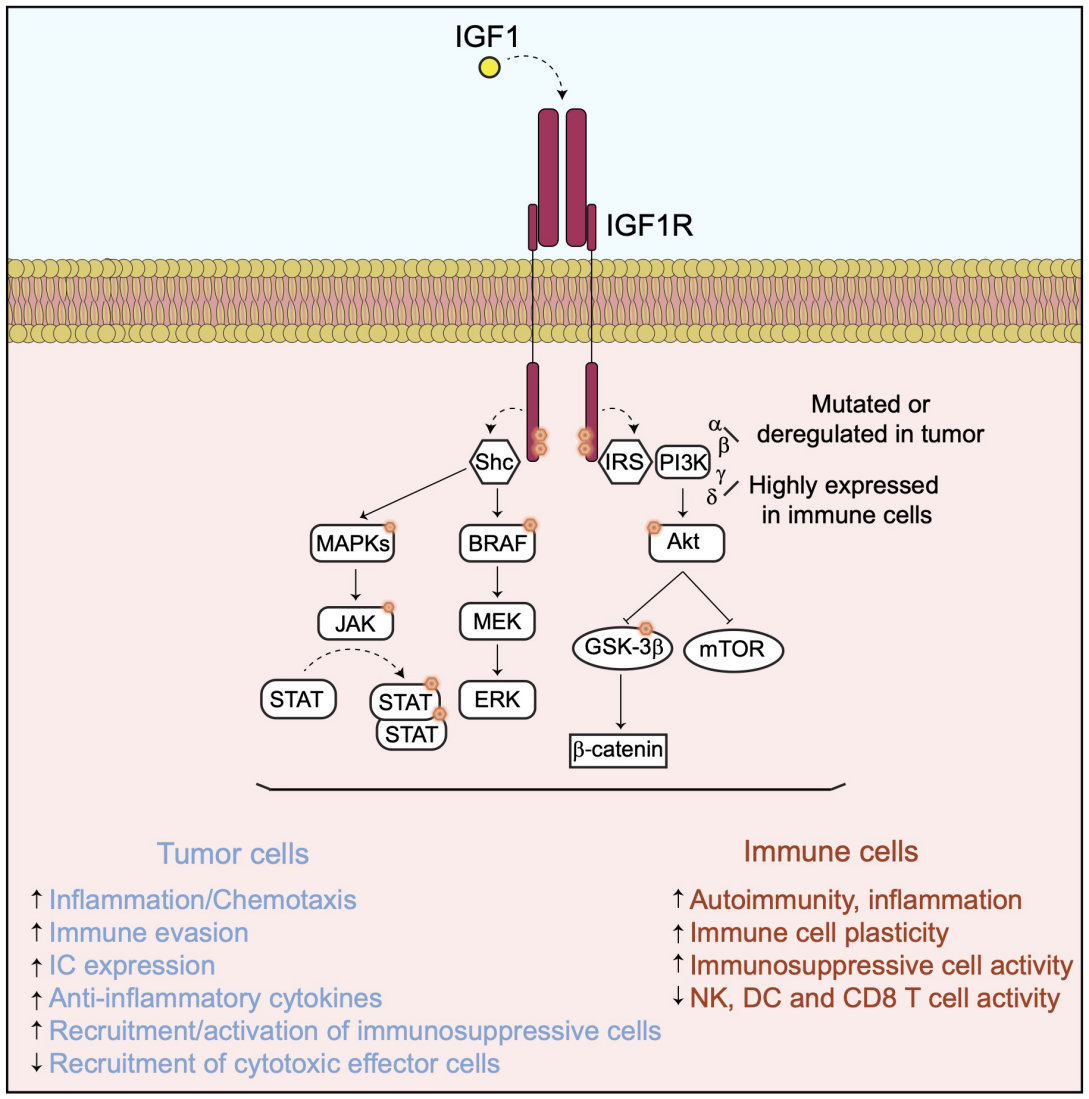
The IGF1/IGF1R axis and its downstream signaling pathway. In presence of IGF1, IGF1R activates downstream signaling pathways implicated in cancer cell proliferation and survival as well as in immunomodulation.

Once activated through interaction with IGF1, IGF1R recruits several docking or adaptor proteins required for the transduction of multiple downstream cell signaling pathways (**Figure 1**).

The recruitment and phosphorylation of the insulin receptor substrate (IRS) by IGF1R leads to the activation of different PI3Ks, including the alpha, beta, gamma and delta isoforms and to their downstream signaling pathways (6–10). All these PI3K isoforms promote tumor cell survival, proliferation and metastasis by regulating different processes including tumor cell metabolism, angiogenesis and by modulating the TME and immunity, mainly via the activation of the AKT/mTOR and the GSK3β/beta-catenin pathways (11) (**Figure 1**). Resistance to PI3K inhibition in cancer cells occurs via activation of different compensatory mechanisms (12) or adaptive responses, including the activation of the IGF1R pathway (12–14). For instance, resistance to PI3K delta inhibition in a chronic model of lymphocytic leukemia is linked to the upregulation of the IGF1R expression resulting in an enhancement of the MAPK pathway (15). In addition to their direct implication in tumor progression, PI3Ks are also required for the regulation of a broad range of immune cell functions implicated in autoimmunity and inflammation, which may vary depending on their isoform specificities (16, 17). Indeed, while the alpha and beta isoforms are ubiquitously expressed and found either mutated and/or activity deregulated in many tumor types, the PI3K gamma and delta are highly expressed in cells of the immune system, though not exclusively. The activation of PI3K delta in response to inflammation plays a critical role in chemotaxis and recruitment of immunosuppressive cells, including regulatory T cells (T-regs) and myeloid-derived suppressor cells (MDSC) (18), while PI3K gamma controls the plasticity of the tumor-associated macrophages toward an immunosuppressive phenotype (19). More recently, the PI3K alpha activation in tumor cells has been associated with immune evasion by promoting a myeloid tumor suppressor microenvironment and decreasing the recruitment of cytotoxic CD8^+^ T cells (20). Conversely, PI3K inhibition promotes anti-tumor immunity by enhancing directly the activity, the recruitment and memory of cytotoxic CD8^+^ T cells and Natural Killer (NK) cells *in vivo* (21, 22). These observations have led to the development of several selective inhibitors of the different PI3K isoforms, which are evaluated in the clinic for different cancer types, either as single agent or in combination with other drugs, including antibodies directed against IC molecules (23).

Other docking molecules are recruited to IGF1R when activated, such as SHC domain proteins which are mainly involved in the activation of the BRAF/MAPK and the JAK/STATs signaling pathways, both important regulators of tumor progression and immunomodulation (24–27) (**Figure 1**). Therefore, the pro-tumor function of IGF1R could be largely mediated by these downstream signaling cascades. Indeed, BRAF and MEK co-inhibition has been shown to counteract the immunosuppressive function of the oncogenic mutant BRAF V600E and to enhance adoptive cell transfer immunotherapy in a mouse model of BRAF V600E-driven melanoma (28). MEK inhibition in RAS-driven tumors enhances the recruitment of cytotoxic CD8^+^ T cells but impairs their intrinsic activation in a reversible manner (29). Also, MEK inhibition suppresses directly the immunosuppressive activities of macrophages, T-reg and MDSC cells (30) and enhances the anti-tumor efficacy of chimeric antigen receptor (CAR) modified T cells (31) as well as of antibodies targeting the inhibitory IC molecules such as PD-1, PD-L1 and CTLA4 (32). Activation of the JAK/STAT3 signaling, together with co-stimulatory pathways, has a profound immunosuppressive activity by inducing the tumor cell expression of genes including PD-L1 (33, 34) and IDO1 (35), anti-inflammatory cytokines, such as IL-10 or TGF beta, and the angiogenic factor VEGF (27). STAT3 modulation acts directly or indirectly by decreasing dendritic cell (DC) activity (36), inhibiting the activation of cytotoxic CD8^+^ T cells (37) and NK cells (38), as well as stimulating the recruitment and differentiation of immunosuppressive macrophages (39), T-reg (40–42) and MDSC cells (43) in the TME.

Altogether, these observations indicate that IGF1R is at the apex of several signaling pathways directly controlling tumor cell proliferation and modulating the immune TME (**Figure 1**).

## 3 IGF1/IGF1R axis and the tumor immune microenvironment

The TME facilitates tumor progression in part by maintaining an immunosuppressive state, impairing anti-tumor immunity. Maintenance of this immunosuppressive state occurs via different mechanisms, leading to the modulation of the activity and recruitment into the TME of different cell populations of the immune system, including T cells, NK cells, DCs, macrophages, neutrophils and MDSCs (3). Among these mechanisms, growing evidence indicates that the IGF1/IGF1R axis plays a key role in regulating the activity of all these immune cell populations.

### 3.1 IGF1R signaling and T cells

In physiological conditions, IGF1R is expressed at the surface of T cells and its activation appears to be important for the regulation of their differentiation and function. For instance, in the thymus, inhibition of the IGF1R pathway induces a blockade of T cell differentiation at the CD4^-^CD8^-^ stage (44). Moreover, IGF1R expression is higher in naïve CD45RA^+^ T cells than in the memory CD45RO^+^ T cell sub-populations (45), indicating an involvement of the IGF1R pathway in T cell development and differentiation.

In addition to its role in development and differentiation, the IGF1R pathway is required at the early stage of T cell activation. Indeed, IGF1R is expressed in resting and activated T cells, and its interaction with IGF1 enhances the proliferation and chemotaxis of PHA-activated T cells (46). Furthermore, Johnson E.W. et al. (47) observed an early and transient upregulation of IGF1R protein expression in circulating T cells activated by anti-CD3 antibody. This transient IGF1R expression correlated with an enhancement of T cell proliferation. In addition, IGF1 increases the transcription of some early markers of T cell activation such as IL-2 and CD25, without affecting the expression of the co-stimulatory CD69 molecule (48).

The function of the IGF1R pathway has been investigated more in-depth for the immunosuppressive T-reg cell subset. T-regs express IGF1R at their cell surface and IGF1 stimulates their activity and proliferation (49). While inhibiting T-reg immunosuppressive functions in tumors might be essential to enhance anti-cancer immunotherapy, most of the functional studies regarding IGF1/IGF1R axis have been performed to boost the function of T-reg activity in the treatment of autoimmune diseases such as type-I diabetes, dermatitis and multiple sclerosis (50). For instance, Bilbao D. et al. demonstrated that IGF1 induces the expression of a gene set associated with T-reg cell proliferation *in vitro* and halted the progression of type 1 diabetes in an *in vivo* mouse model. IGF1R activation was also directly implicated in the amplitude and quality of the T-reg immunosuppressive response (49). More recently, Shapiro M.R. et al. (51) showed that IGF1 synergizes with IL-2 to stimulate the proliferation of T-regs in type 1 diabetes. This synergistic effect is mediated via an enhanced and sustained activation of the STAT5 pathway, which is only transiently activated in T-regs upon IL-2 treatment alone. In addition, they observed that IGF1 and IL-2 co-treatment increases the expression of the IL-2 receptor alpha chain, CD25, explaining the enhancement of T-reg cell proliferation (51). Moreover, IGF1 has been shown to increase the number of T-regs in the area affected by allergic contact dermatitis, an aberrant hyper-inflammatory immune response, and to control the production of anti-inflammatory cytokines such as IL-10 (52). However, the IGF1R pathway has been demonstrated to favor the T-helper-17 cell differentiation over that of the T-regs in multiple sclerosis. This effect was mediated through the activation of the AKT/mTOR pathway and up-regulation of the aerobic glycolysis pathway (53).

In cancer, few studies report the role of the IGF1R pathway in modulating T cell activity. In a mouse model of hepatocellular carcinoma, Huang Y. et al. have identified two intrahepatic subsets of T-regs based on the expression level of IGF1R. IGF1R^high^ T-regs had increased PI3K/AKT/mTOR signaling pathway, were metabolically more active, more proliferative, and were producing more immunosuppressive cytokines than IGF1R^low^ T-regs (54). Ajona D. et al. have subsequently demonstrated that co-targeting PD-1 and IGF1R resulted in a significant decrease in the number of T-regs and an increase in intratumoral cytotoxic CD8^+^ T cells leading to an improvement of anti-tumor efficacy in an *in vivo* mouse model of lung cancer (55). In a similar study, Wu Q. et al. have observed that the IGF1/IGF1R axis inhibition enhanced the efficacy of immunogenic chemotherapy, which correlated with an increase in tumor infiltrating effector T cells and a decrease in T-regs, in breast tumor mouse models (56, 57).

**Figure 2:**
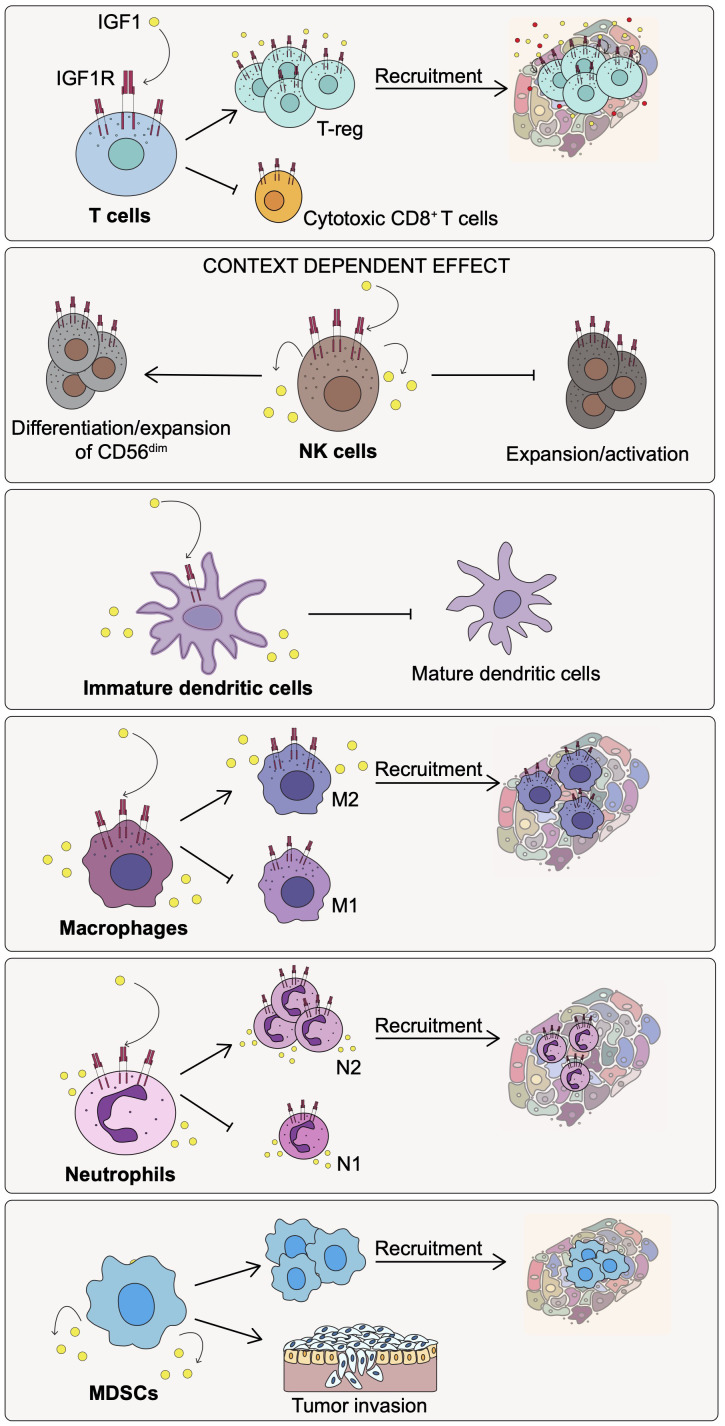
Effect of IGF1/IGF1R pathway activation on the immune cell populations of the TME. Activation of the IGF1/IGF1R axis affects differently the indicated immune cell subtypes promoting immunosuppression and cancer progression.

Altogether, the IGF1/IGF1R axis plays a role in T cells development and differentiation as well as in the early step of T cell activation. In addition, the IGF1/IGF1R pathway appears to be a key player in enhancing the immunosuppressive function of T-regs and impairing the recruitment of cytotoxic CD8^+^ T cells at the tumor site (**Figure 2**).

### 3.2 IGF1R signaling and Natural Killer cells

Natural Killer (NK) cells are a subtype of lymphocytes involved in the innate immune response. In contrast to T cells, which recognize specific antigens expressed at the surface of target cells, NK cells identify virus-infected, damaged and malignant cells using activating and inhibitory receptors present at their surface. NK cells secrete IGF1 and express IGF1R at their surface. However, the role of the IGF1/IGF1R axis in regulating the differentiation, proliferation and cytotoxic activity of NK cells is subject to debate.

On one side and in normal condition, IGF1 promotes the differentiation and the expansion of the more cytotoxic effector CD56^dim^ NK cell sub-set, to the detriment of the less cytotoxic CD56^bright^ NK cells, suggesting a role for IGF1 in NK cell development and cell fate (58). Also, the CD56^dim^ NK cells were found to express a higher level of IGF1 when compared to the CD56^bright^ subtype in the uterine decidua (59). On the other side and in the context of autoimmune diseases such as systemic lupus erythematosus, IGF1 expression levels are inversely correlated with the number of specific immune cell types including NK cells. Also, Shi J.W. et al. demonstrated that the high IGF1 expression by the decidual stroma cells during pregnancy is mediated via the upregulation of WISP2, a member of the WNT1 signaling pathway. The release of IGF1 by these decidual stroma cells causes a downregulation of the cytotoxic activity of NK cells through activation of the IGF1R pathway (60).

The same controversy appears also to be observed in tumors. In hepatocellular carcinoma, the forced overexpression of a specific miRNA, miR-615-5p, which represses IGF1R expression, decreases the number of CD56^dim^ and increases the number of the CD56^bright^ NK cells (61). In the same tumor model, the cytotoxic activity of NK cells was enhanced following the over-expression of miR-486-5p, an inducer of IGF1 protein expression (62). In addition, derivatives of the natural substance ginseng are reported to increase the cytotoxicity of NK cells against lymphoma cells *in vitro*, through the enhancing of an IGF1-dependent mechanism (63). On the contrary, however, inhibition of the IGF1R signaling pathway using an IGF1R-specific antibody is reported to enhance NK cell expansion following their activation and to maintain their potent cytotoxic activity *in vitro* against Ewing sarcoma (64).

Altogether, these results suggest that the role played by the IGF1/IGF1R axis on NK cells may be context-dependent (**Figure 2**). It may relate to the differentiation state of NK cells but also to the composition of the surrounding environment in terms of immune and non-immune cells, and associated factors as well as to the IGF1R co-signaling pathways involved.

### 3.3 IGF1R signaling and dendritic cells

Dendritic cells (DCs) are specialized in presenting antigens to T cells for the initiation of the immune response and tolerance (65). DCs express IGF1R and its activation appears to be linked to the phenotypic and functional maturation of the DCs. However, depending on the context, the IGF1/IGF1R axis may have an opposite role.

In normal condition, IGF1 enhances the phagocytic activity of bone-marrow-derived DCs but at the same time decreases their LPS-induced TNFα release through the activation of the PI3K/AKT pathway (66). Furthermore, Liu E. et al. have shown that IGF1 promotes the maturation of cord blood monocyte-derived DCs (MoDCs) by enhancing the expression of specific DC markers and of the major histocompatibility complex (MHC) class II molecules (67). However, in contrast to the previous study, they observed that IGF1 increases the production of TNF alpha and the survival of these MoDCs. Both PI3K and MEK activations by IGF1 were involved in the maturation and survival of MoDCs.

In tumors however, the IGF1/IGF1R axis appears to play an opposite function. DCs infiltrated in the TME are particularly vulnerable to oncogenic stimuli (65), such as those induced by IGF1. Indeed, Huang C.T. et al. (68) demonstrated that in an advanced stage model of ovarian cancer, IGF1 suppresses DC maturation and their antigen-presenting capability, resulting in a failure to activate the antigen-specific CD8^+^ T cells and, thereby, the instauration of the primary immune response and immunologic memory. Moreover, DCs incubated with IGF1 secreted higher levels of cytokines such as TNFα and IL-10. These IGF1 effects were mediated through a decrease of ERK and P38 signaling and were reverted by pharmacological inhibition of the IGF1R kinase activity. Similarly, Somri-Gannam L. et al. (69) have shown that there is an inverse correlation between the expression of IGF1R and CD1c, a marker of mature DCs, in epithelial ovarian cancer samples. In addition, they show that specific inhibition of IGF1R in DCs decreases ovarian cancer cell migration.

In summary, the function of the IGF1R pathway in DCs may be dependent on the surrounding environment, the pathological or physiological conditions, and on the signaling pathways involved. In cancer, the IGF1/IGF1R axis appears to play a role in modulating DC activity toward an immature state with immunosuppressive and tumor-promoting activities (**Figure 2**).

### 3.4 IGF1R signaling and macrophages

Macrophages are important immune cells for the activation of the innate and adaptive immune responses through their ability to release pro-inflammatory cytokines. In the presence of various stimuli, macrophages can acquire different phenotypes and functions. Pro-inflammatory macrophages, also called M1, are polarized upon lipopolysaccharide (LPS) stimulation, while anti-inflammatory macrophages, called M2, are induced by IL-4 and IL-13 (70). Macrophages synthetize and release high levels of IGF1 and express IGF1R at their cell surface. Activation of the IGF1R pathway in macrophages appears to play a pivotal role in the polarization of the macrophages from pro-inflammatory, M1, to an anti-inflammatory, M2, phenotypes.

Spadaro O. et al. (71) demonstrated that M2-macrophages express high levels of IGF1 when compared to the M1 subtype, and that the IGF1R signaling sustains the activity of M2-macrophages in response to immuno-metabolic challenges, such as high-fat diet-induced obesity in mice. In a macrophage-specific IGF1R-knockout mouse model of atherosclerosis, Higashi Y. et al. (72) have shown that the proinflammatory response was enhanced, as the M1-associated markers were found to be highly expressed on the surface of macrophages. In another study, it was demonstrated that macrophages engineered to express high levels of IGF1 reduced atherosclerosis burden, suggesting an increase in anti-inflammatory M2 macrophages (73). By generating inactivating IGF1 mutation targeted to myeloid cells, Tonkin J. et al. demonstrated that IGF1 has an autocrine role in driving or influencing macrophages toward an M2 phenotype during muscle regeneration following injury (74). Moreover, Barett J.P. et al. have shown that the anti-inflammatory cytokine IL-4 induces an upregulation of IGF1 expression by the macrophages, and that its release is necessary for full activation of bone marrow-derived macrophages with an M2 phenotype (75).

All these studies indicate a crucial role for the IGF1/IGF1R axis in promoting an M2, rather than an M1 phenotype. However, in a different context, Shan X. et al. observed that serine metabolism regulates the polarization of macrophages toward an M1 phenotype via the modulation of the IGF1 pathway (76). This effect was mediated through the activation of the P38/JAK/STAT1 pathway by IGF1. Similarly, Ieronymaki E. et al. (77) have shown that macrophages lacking IGF1R are resistant to insulin and that insulin-resistance promotes an M2-like phenotype.

Altogether, this evidence indicates a role for the IGF1/IGF1R axis in regulating M1/M2 macrophage plasticity, which may depend on the context, the disease and the environment surrounding the macrophages.

The presence of macrophages with a M2-like phenotype in the TME contributes to immunosuppression and tumor progression while the M1-like macrophages possess anti-cancer activity. The studies have demonstrated the role of the IGF1/IGF1R pathway in promoting an M2-like macrophage phenotype. In a mouse model of glioma, the TME has been shown to promote resistance to long-term inhibition of the colony-stimulating factor-1 receptor. This effect was dependent on an increase in the IGF1R/PI3K/AKT signaling pathway by the tumor cells which was induced by M2-like macrophage-derived IGF1 present in the TME (78). In breast cancer, the IGF1/IGF1R axis activation correlated with the level of pro-tumoral M2-like tumor-associated macrophages (TAM) which, together with tumor-associated fibroblasts, was the main source of IGF1 (79). Similarly, the M2-like TAM contributes to thyroid cancer stemness and metastasis via the secretion of high amounts of IGF1 (80). In addition, the TAM-derived IGF1 is directly implicated in the growth and migration of ovarian cancer cells (81). Zhang W. et al. (82) have shown that the signaling through EGFR, another growth factor receptor expressed at the surface of tumor cells, contributes to colon cancer progression via an IGF1-mediated M2 macrophage polarization mechanism. More recently, Alfaro-Armedo E. et al. (83) have used an IGF1R deficient mouse model to demonstrate the role of the IGF1R pathway in facilitating lung metastasis implantation and progression. In this mouse model, tumor burden, vascularization and inflammation, as well as the number of M2-like macrophages and intra-tumoral T-regs were reduced (83).

Overall, these observations suggest that IGF1/IGF1R axis in cancer plays a critical role in promoting the recruitment and the maintenance of immunosuppressive macrophages with an M2 phenotype in the TME, thereby, contributing to the inhibition of anti-cancer immunity and enhancement of tumor progression (**Figure 2**).

### 3.5 IGF1R signaling in neutrophils and MDSCs

Neutrophils are a set of highly heterogeneous cell populations with multifaceted functions (84). Similarly to macrophages, they may have pro- (N1) or anti-inflammatory (N2) phenotypes, and the IGF1 pathway has been demonstrated to play a pivotal role in regulating their N1/N2 plasticity. For instance, in myocardial infarction, Nederlof R. et al. (85) demonstrate that the IGF1/IGF1R axis attenuates the pro-inflammatory phenotype of neutrophils by upregulating the expression of anti-inflammatory genes via the activation of the JAK2/STAT6 pathway. In different lung injury models, such as those induced by bleomycin, smoke or hyperoxia, IGF1R deficiency provokes a decrease in the recruitment of immunosuppressive neutrophils at the site of injury (86, 87). Moreover, neutrophils express high levels of IGF1R, and its blockade decreases the number of circulating immunosuppressive neutrophils (88).

MDSC arise from bone marrow precursors that differentiate in immature myeloid cells (IMC) that further give rise to the polymorphonuclear (PMN)- or monocytic (M)-MDSC cell subsets. While in physiological conditions, myelopoiesis generates neutrophils, monocytes and DCs, during pathological conditions characterized by a high status of inflammation, IMC differentiate into MDSCs with a potent immunosuppressive activity (89). MDSCs are particularly abundant in the TME and are involved in cancer progression and metastasis (84). The role of the IGF1/IGF1R axis in directly modulating MDSCs activity in cancer, has not yet been deeply investigated. Only one report shows that the release of different factors by MDSCs, including IGF1, promotes the invasive phenotype of carcinoma cells (90). Although more work remains to be done to understand the critical role of the IGF1R/IGF1 axis in regulating MDSCs activity, based on the above observations we can speculate that IGF1/IGF1R pathway may have a role in recruiting MDSCs at the tumor site, and also in the differentiation of their immunosuppressive and, therefore, pro-tumorigenic activities (**Figure 2**).

## 4 Targeting IGF1R and the immuno-oncotherapy perspective

Several strategies have been developed to target IGF1R in cancer. Among them are antisense oligonucleotides to downregulate gene expression, DNA, peptides or cell vaccines to elicit antibody responses, small molecules to block kinase activity and antibodies to impair receptor-ligand interaction. Many have been tested in different pre-clinical settings leading, for some of them, to the opening of clinical trials for different tumor types (91). However, and as recently reviewed by Jentzsch V. et al., IGF1/IGF1R targeted agents underwent over 183 clinical trials and none of them have yet been approved for oncological diseases (92). While partial responses have been observed with some of the IGF1R inhibitors, either as single agent or in combination with chemotherapeutics and targeted agents, no significant efficacy and patient benefit has yet been seen in clinic so far (93). Nonetheless, the emerging role of the IGF1R pathway inhibition in regulating immunomodulation and boosting anti-tumor immunity appears to open new therapeutic opportunities for the IGF1R targeting agents.

Antisense approaches to target IGF1R expression have been in development for a long time. For instance, the administration of a siRNA molecule modified to increase its *in vivo* delivery and stability, induces a delay in tumor growth, accompanied by an increase in the release of TNFα and IFNγ pro-inflammatory cytokines in a xenograft model of breast cancer but has never been used in the clinic (94). Targeting IGF1R through an antisense oligodeoxynucleotide (IMV-001) has proven to be an excellent strategy to increase the immunogenicity of cancer cells as demonstrated by the use of the IGV001 cancer vaccine (95, 96). This personalized cancer cell-based vaccine implies the incubation of IMV-001 oligonucleotide with autologous cancer cells in a biodiffusion device *ex vivo*. This biodiffusion device is subsequently irradiated and then implanted in the abdomen of a living specimen. In a mouse model of glioma, IGV001 stimulates the immune system by inducing the release of immunogenic cell death (ICD)-associated molecules such as ATP and HMGB1. Also, Cultrara C. et al. (95) observed an increase in the number of activated antigen-presenting DCs as well as of effector and memory T cells at lymph nodes and demonstrated the efficacy benefit of combining IGV001 with antibodies targeting PD-1. IGV001 has proven to be well tolerated and to show promising signs, although limited, of efficacy in phase 1 clinical trial (NCT02507583) (97, 98), leading to the recent opening of a phase 2b for patients affected by glioblastoma (NCT04485949).

Vaccines using peptides or DNA construct coding for IGF1R peptides have been shown to induce antibody production and to elicit an active immune response against IGF1R. One advantage of these vaccines is that multiple onco-drivers can be targeted simultaneously (99). For instance, WOKVAC, a DNA-based vaccine that encodes for multiple epitopes derived from IGF1R, IGFBP2, a molecule involved in IGF1 stabilization, and HER2, has been shown to activate a humoral immune response in patients affected by breast cancer and is entering a phase 2 clinical trial in combination with chemotherapeutic agents (NCT04329065).

Small molecules targeting IGF1R, including picropodophyllin (PPP) as well as the investigational drugs, BMS754807 and linsitinib, have been shown to increase the anti-tumor efficacy of chimeric antigen receptor (CAR) engineered T cells (100, 101). In particular, linsitinib induces tumor cell death through IGF1R pathway inhibition and, at the same time, decreases the expression of CAR T-cell exhaustion markers and increases their central memory profiles without interfering with their cytotoxic activity *in vitro*. Moreover, linsitinib in combination with CAR T-cells targeting the disialoganglioside GD2 demonstrates a sustained anti-tumor efficacy in an *in vivo* model of pediatric diffuse midline glioma (100). The effect of linsitinib on CAR T-cell phenotypes is likely to be mediated via a linsitinib off-target, as CAR T-cells do not express the IGF1R receptor. In another study, linsitinib and PPP treatments have been found to increase the ICD induced by immunogenic chemotherapeutic agents, such as oxaliplatin. In an *in vivo* model of triple-negative breast cancer, both PPP and linsitinib in combination with oxaliplatin increase macro-autophagy and ATP release by the dying tumor cells resulting in DC activation, recruitment of cytotoxic CD8^+^ T cells, and decrease of T-regs number at the tumor site (57). Moreover, the synergistic anti-tumor activity of an anti-PD-1 antibody in combination with PB-020, a novel PPP-derived small-molecule inhibitor of IGF1R with improved pharmacokinetic properties, has been demonstrated in a mouse model of colorectal cancer (102).

As mentioned above, several monoclonal antibodies targeting IGF1R or IGF1 proteins have been tested in the clinic for different diseases, including cancer. While demonstrating no clinical benefit in terms of efficacy when used as monotherapy, these antibodies are currently being tested in combination with other targeted and chemotherapeutic agents (91). To our knowledge, their uses in combination with other immunotherapeutic drugs have not been investigated yet.

It is also to note that CAR T-cells engineered to target the IGF1R protein expressed at the surface of tumor cells have been developed and demonstrated to be effective in osteosarcoma xenograft models (103). However, their safety and efficacy remain to be demonstrated in the clinic.

Collectively, these pre-clinical and clinical results strongly support the development of therapeutic strategies targeting the IGF1/IGF1R axis to boost anti-cancer immunity and to potentially improve the efficacy of current immuno-oncotherapeutic approaches.

## 5 Conclusions

**Figure 3:**
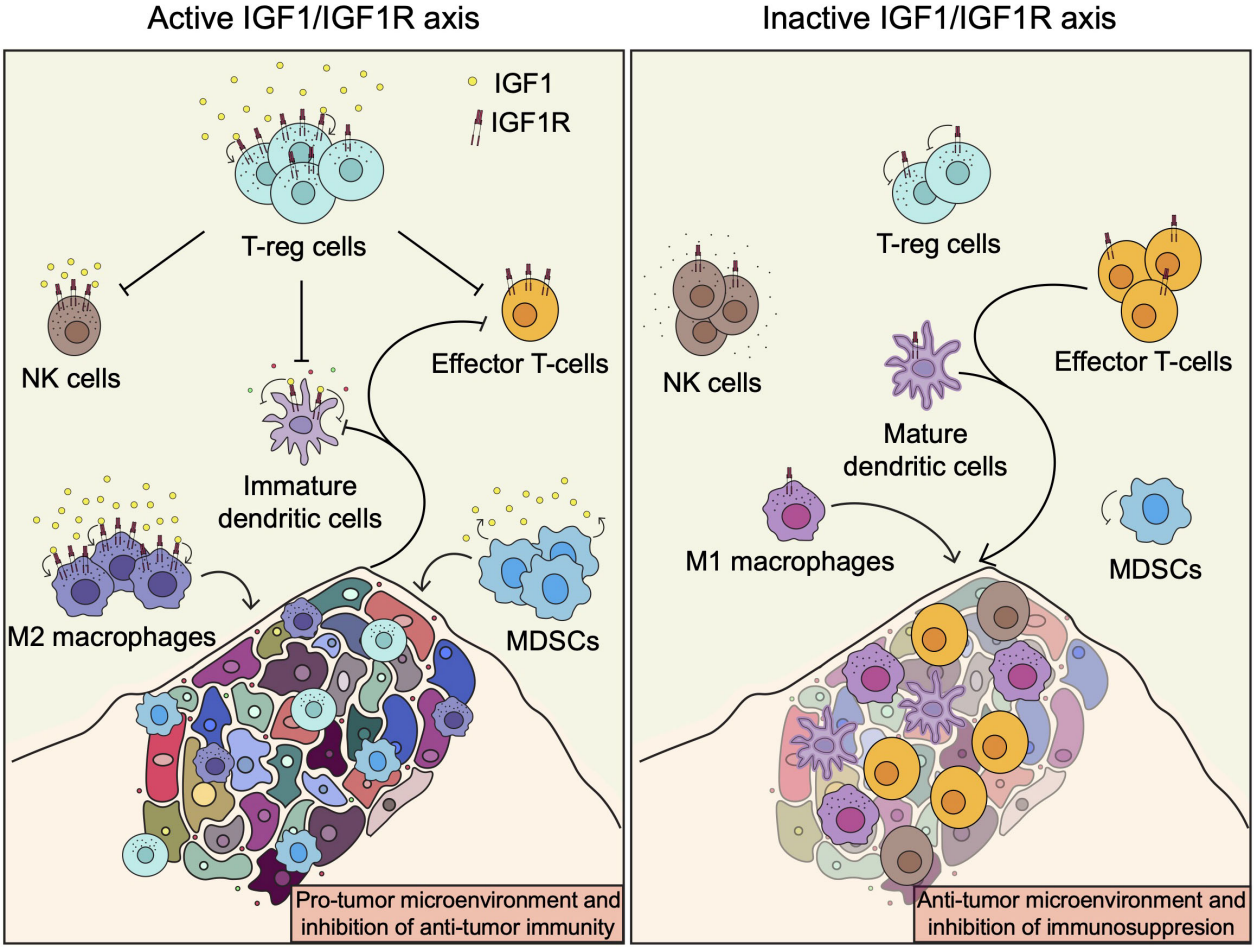
The IGF1/IGF1R axis and the tumor immune microenvironment. Induction of the IGF1R pathway enhances recruitment and activation of immune cells involved in immunosuppression, such as T-regs, M2 macrophages and MDSCs, leading to inhibition of anti-tumor immunity. In contrast, inhibition of the IGF1R pathway or abscence of IGF1 enhance anti-tumor immunity by promoting the recruitment and activation of M1 macrophages and DCs as well as effector cytotoxic CD8^+^ T cells and potentially NK cells.

The above observations indicate that IGF1R has immuno-modulatory potential and that its targeting may be exploited to potentiate the intrinsic anti-cancer activity of the immune system and could be used in combination with different immunotherapeutic agents to improve cancer treatment. Inhibition of the IGF1/IGF1R axis will decrease the number of T-regs, M2 macrophages and MDSCs, and enhance the recruitment and activity in the TME of M1 macrophages and DCs as well as effector cytotoxic CD8^+^ T cells and potentially NK cells (**Figure 3**). Therefore, targeting IGF1R will have a beneficial dual mode of action by inducing tumor cell death and, at the same time, enhancing the anti-tumor immune cell responses. While most of the strategies to target the IGF1/IGF1R so far have shown no or limited efficacy in the clinic, combining IGF1R inhibitors with anticancer immunotherapeutic or other targeted immunomodulatory drugs will certainly be a valuable strategy to overcome or prevent immunosupression and improve the treatment of patients affected by cancer. The promising results obtained from the IGV001 and VOKVAC phase 1 clinical trials are the demonstration that manipulating the tumor immune microenvironment through targeting the IGF1/IGF1R axis is safe and feasible. Ongoing investigations will determined if these strategies can translate into patient benefits.

More in-depth and systematic studies on the pleiotropic and context-dependent role played by the IGF1/IGF1R pathway in modulating immunity with regards to specific tumor types will also help in defining, in a near future, the best IGF1R inhibitor/immuno-oncotherapeutic combination strategies to treat and ultimately cure cancer patients.

## Author contributions

MP: Writing – original draft. VS: Writing – original draft. SDA: Writing – original draft. LLP: Writing – original draft. MC: Writing – original draft. VF: Writing – original draft. NT: Writing – original draft. PV: Writing – original draft. MV: Writing – original draft. DF: Writing – review & editing. EDB: Writing – original draft, Writing – review & editing.
